# Association of cerebrospinal fluid anti-ribosomal P protein antibodies with diffuse psychiatric/neuropsychological syndromes in systemic lupus erythematosus

**DOI:** 10.1186/ar2184

**Published:** 2007-05-02

**Authors:** Shunsei Hirohata, Yoshiyuki Arinuma, Maki Takayama, Taku Yoshio

**Affiliations:** 1 Department of Rheumatology and Infectious Disease, Kitasato University School of Medicine, 1-15-1 Kitasato, Sagamihara, Kanagawa 228-8555, Japan; 2 Department of Internal Medicine, Teikyo University School of Medicine, 2-11-1 Kaga, Itabashi-ku, Tokyo 173-8605, Japan; 3 Division of Rheumatology and Clinical Immunology, Jichi Medical University, 3311-1 Yakushiji, Shimotsuke, Tochigi 329-0498, Japan

## Abstract

We explored the relationship of antibodies to the whole ribosomal P proteins (P0, P1, and P2) in cerebrospinal fluid (CSF) with diffuse psychiatric/neuropsychological syndromes in systemic lupus erythematosus (SLE). CSF samples were obtained from 71 SLE patients (52 patients with diffuse psychiatric/neuropsychological syndromes [diffuse NP-SLE] and 19 patients with neurological syndromes or peripheral neuropathy [focal NP-SLE]) as well as from 24 patients with non-inflammatory neurological disease. Immunoglobulin G (IgG) antibodies to the C-terminal 22-amino acid ribosomal P synthetic peptide (anti-P_C22_) and those to purified bovine ribosomal P proteins (P0, P1, and P2) (anti-whole P) were determined by enzyme-linked immunosorbent assay; affinity-purified IgG anti-P_C22 _were used as the standard. The concentrations of antibodies to epitopes other than the C-terminal 22 amino acids of ribosomal P proteins were calculated by subtracting anti-P_C22 _from anti-whole P (anti-P_EX.C22_). CSF anti-whole P levels were significantly elevated in diffuse NP-SLE compared with focal NP-SLE or control patients. By contrast, there were no significant differences in CSF anti-P_C22 _levels among the three groups. Of note, CSF anti-P_EX.C22 _levels were significantly elevated in diffuse NP-SLE compared with the other two groups. CSF anti-P_EX.C22 _levels were not significantly correlated with CSF anti-P_C22 _levels, but with CSF antibodies against the recombinant ribosomal P0 protein lacking the C-terminal 22 amino acids (C22-depleted rP0). Moreover, levels of CSF anti-P_EX.C22 _or CSF anti-C22-depleted rP0, but not CSF anti-P_C22_, were significantly correlated with CSF anti-neuronal cell antibodies (anti-N). These results indicate that CSF IgG antibodies to the epitopes other than the C-terminal 22 amino acids of ribosomal P proteins, which might contain one of the major targets of CSF anti-N, are associated with the development of diffuse NP-SLE.

## Introduction

Central nervous system (CNS) involvement is a relatively common and serious complication of systemic lupus erythematosus (SLE) [1,2]. Previous studies have demonstrated the association of serum antibodies directed against the C-terminal 22-amino acid sequences of ribosomal P protein (anti-P_C22_) with CNS involvement in patients with SLE (neuropsychiatric SLE [NP-SLE]), especially diffuse psychiatric/neuropsychological syndromes (diffuse NP-SLE) [3-5]. However, the mechanism by which serum anti-P_C22 _leads to the development of diffuse NP-SLE has not yet been elucidated. In fact, the role of anti-P_C22 _in the cerebrospinal fluid (CSF) in the pathogenesis with diffuse NP-SLE or even their presence in the CSF remains uncertain. Thus, Golombek and colleagues [6] detected the presence of CSF anti-P_C22 _in all four of the patients with lupus psychosis in their studies, whereas others did not [3,4,7].

On the other hand, autoantibodies, which react with the neuronal cell lines or brain tissue, have been reported in the sera of patients with NP-SLE [8-10]. However, they have been shown to be present in SLE patients with no clinical evidence of CNS involvement [10]. In fact, in a cross-sectional study of SLE patients, no significant association was found between serum lymphocyte/brain cross-reacting antibodies and NP-SLE (present in 32% of cases with NP-SLE and 23% of those without NP-SLE) [10]. Of note, using a radioimmunoassay with the SK-N-SH neuroblastoma cell as a target, Bluestein and colleagues [11] demonstrated that immunoglobulin G (IgG) anti-neuronal cell antibodies (anti-N) were present in much higher concentrations in the CSF from patients with active NP-SLE than in the CSF from SLE patients without active CNS involvement. Using a cell enzyme-linked immunosorbent assay (ELISA) with SK-N-MC neuroblastoma cell lines fixed with paraformaldehyde, we also confirmed that CSF IgG anti-N levels were significantly elevated in patients with diffuse NP-SLE compared with those in SLE patients without diffuse NP-SLE [7]. However, the fine epitopes to which CSF anti-N were directed have not yet been delineated.

The presence of the immunodominant C-terminal epitope of ribosomal P proteins was demonstrated to be present on the surface of human neuroblastoma cells [12]. However, CSF anti-P_C22 _could be detected in only a fraction of patients with diffuse NP-SLE, whereas almost all the patients with diffuse NP-SLE expressed CSF anti-N [7]. Of note, previous studies also demonstrated the presence of a 38-kDa protein that is closely related to, or identical with, ribosomal P0 protein in purified human plasma membranes [12]. In addition, it has been shown that autoantibodies directed against the ribosomal P proteins are not only directed against the common C-terminal 22 amino acids, but against the N-terminal sequence of the ribosomal P2 or P1 proteins [13]. In fact, recent studies have revealed that measurement of CSF IgG anti-ribosomal P protein antibodies with Western blotting using purified ribosomes, containing whole ribosomal P0, P1, and P2 proteins, was more sensitive [14]. Because ribosomal P0 protein contains epitopes other than the C-terminal 22 amino acids, it is possible that CSF from patients with diffuse NP-SLE contains antibodies to such epitopes. The current studies, therefore, were carried out to compare the CSF levels of antibodies to the whole ribosomal P proteins (anti-whole P) in patients with diffuse NP-SLE and in patients with focal NP-SLE or non-SLE non-inflammatory neurological disorders.

## Materials and methods

### Patients and samples

One hundred and three patients with SLE were included in the present study. All patients fulfilled the American College of Rheumatology (ACR) 1982 revised criteria for the classification of SLE [15]. Of the 103 patients with SLE, 52 showed diffuse psychiatric/neurological syndromes (diffuse NP-SLE) according to the 1999 ACR definition of NP-SLE [16], 19 patients showed CNS manifestations other than diffuse NP-SLE (focal NP-SLE), and 32 patients showed no CNS manifestations (non-CNS SLE). Ten of the 52 patients with diffuse NP-SLE also presented seizures. Because of the difficulties in confirming the neurological diagnosis and in assigning the cause to SLE, we defined NP-SLE as (a) the presence of neuropsychiatric manifestations and (b) the elevation of CSF Ig indices [17,18] and/or the elevation of CSF interleukin-6 (IL-6) levels [19]. Thus, the 52 patients all showed increased CSF Ig indices and/or CSF IL-6 in the present study. In addition, 24 patients with non-SLE non-inflammatory neurological diseases (9 cerebrovascular diseases, 8 cervical spondylosis, 4 degenerative diseases, 2 diabetic neuropathy, and 1 epilepsy) were studied as a control. The 127 patients all gave informed consent, and the study was approved by the institutional ethical committee of Teikyo University School of Medicine (Tokyo). The detail and demographic features of the 127 patients are shown in Table 1. CSF specimens were obtained by a lumbar puncture when the patients showed active disease. These samples were kept frozen at -20°C until assayed. All assays were performed without knowledge of the diagnosis or clinical presentations.

**Table 1:** Profiles of the patients studied

Diagnosis	Number of patients	Gender (male/female)	Age in years (mean ± SD)
SLE	103		
Diffuse NP-SLE	52	4/48	37.8 ± 14.2
Acute confusional state	20		
Anxiety disorder	3		
Cognitive dysfunction	10^a^		
Mood disorder	12^b^		
Psychosis	7		
Focal NP-SLE	19	2/17	39.0 ± 14.8
Cerebrovascular disease	6		
Headache	2		
Movement disorder	1		
Seizure disorder	6		
Polyneuropathy	4		
Non-CNS SLE	32	3/29	42.7 ± 13.9
Non-SLE control	24	22/2	48.0 ± 13.7

^a^One patient also presented mood disorder. ^b^One patient also presented cognitive dysfunction. Non-CNS SLE, systemic lupus erythematosus without neuropsychiatric manifestations; NP-SLE, neuropsychiatric systemic lupus erythematosus; SD, standard deviation; SLE, systemic lupus erythematosus.

### Human anti-P_C22 _sera and affinity purification of anti-P_C22_

IgG fractions were purified from the anti-P_C22_-positive sera of SLE patients by means of a protein G-Sepharose 4FF column (Amersham Pharmacia Biotech, now part of GE Healthcare, Little Chalfont, Buckinghamshire, UK). Anti-P_C22 _were purified from the IgG fractions of SLE sera by means of an *N*-hydroxysuccinimide-activated Sepharose HP column (GE Healthcare) coupled with synthetic ribosomal P peptide-human serum albumin (HSA) conjugates as previously described [20]. Anti-P_C22 _thus purified reacted strongly with ribosomal P peptide-HSA conjugates, but not with HSA alone in an ELISA. It was also confirmed on Western blot analysis that purified anti-P_C22 _reacted with native ribosomal P proteins (P0, P1, and P2) (data not shown).

### Measurement of autoantibodies to ribosomal P proteins

Antibodies for the C-terminal 22-amino acid ribosomal P synthetic peptide (anti-P_C22_) in sera and CSF and those for purified whole ribosomal P proteins (anti-whole P) in CSF were determined by specific ELISA using the highly purified synthetic C-terminal 22-amino acid ribosomal P peptide conjugated to HSA as an antigen as previously described [5] and highly purified bovine ribosomal P proteins (P0, P1, and P2) (purity of more than 90%) (Arotec Diagnostics Limited, Wellington, New Zealand). Antibodies for the epitope representing regions of the ribosomal P proteins other than P_C22 _were similarly determined by ELISA using recombinant ribosomal P0 fusion protein lacking the C-terminal 22 amino acids (C22-depleted rP0) as previously described [21].

Briefly, wells of a 96-well microtiter plate were coated with ribosomal P peptide-HSA conjugates at 15 μg/ml or highly purified bovine ribosomal P proteins at 1.0 μg/ml in phosphate-buffered saline (PBS) (pH 7.2) or C22-depleted rP0 at 5 μg/ml in 6 M urea/10 mM Tris-HCl (pH 7.5) with 2 mM 2-mercaptoethanol (coating buffer) at 4°C overnight. Each well was then overcoated with Block Ace (Dainippon Pharmaceutical, Osaka, Japan), diluted 1:4 with PBS. Prior to being added to the antigen-coated wells, serum and CSF samples were usually diluted 1:200 and 1:2, respectively, in PBS containing 1% bovine serum albumin (Miles, now part of Bayer Corp., Emeryville, CA, USA). Bound antibody was detected with peroxidase-conjugated F (ab')_2 _fragments of goat anti-human IgG (MP Biochemicals, Solon, OH, USA). After incubation with substrate solution containing 60 mg of *o*-phenylenediamine and 10 μl of 30% H_2_O_2 _in 100 ml of 0.05 M citrate phosphate buffer (pH 4.8) at 37°C for 30 minutes, the reaction was stopped by addition of 5 N H_2_SO_4_, and the absorbance (optical density) at 492 nm (OD_492_) was read with a two-wavelength microplate photometer (MTP-120; Corona Electric Co., Ltd., Ibaraki, Japan). Determinations of OD_492 _were normalized to affinity-purified anti-P_C22 _such that anti-P_C22 _and anti-whole P activity might be converted to micrograms per milliliter of IgG. Antibodies directed against C22-depleted rP0 (anti-C22-depleted rP0) were expressed by arbitrary unit designation using a standard serum.

Non-specific binding activities to HSA for anti-P_C22 _or those to wells with PBS alone or coating buffer alone for anti-whole P or anti-C22-depleted rP0 were also determined in reference to the standard curves for binding activities to ribosomal P peptide (P_C22_)-HSA conjugates, highly purified ribosomal P proteins, or C22-depleted rP0. The specific anti-P_C22_, anti-whole P, or anti-C22-depleted rP0 activities were thus determined by subtracting the values for the non-specific binding activity from those for binding activity to P_C22_-HSA conjugates or to highly purified ribosomal P proteins or C22-depleted rP0. The intra-assay and interassay variances (coefficient of variation values) for anti-whole P were 13.8% and 15.7%, respectively, and those for anti-P_C22 _were previously described [7].

### Measurement of anti-N

Anti-N in the CSF samples were determined by a cell ELISA using human neuroblastoma cell line SK-N-MC as previously described [7]. Briefly, SK-N-MC cells were seeded at a density of 5 × 10^4 ^per well in wells of a flat-bottomed 96-well tissue culture plate (no. 3596; Costar, now part of Corning Life Sciences, Acton, MA, USA) for 48 hours, after which the cells were fixed with 1% paraformaldehyde in PBS for 5 minutes at 37°C. After three washes with PBS containing 0.05% Tween 20, 50 μl of the appropriately diluted samples or various concentrations of standard sera were added and the plates were incubated for 1 hour at 37°C. Bound IgG anti-N were detected with peroxidase-conjugated F(ab')_2 _fragments of goat anti-human IgG as previously described [7]. Determination of OD_492 _was normalized to standard sera for anti-N obtained from patients with diffuse NP-SLE such that anti-N activity might be converted to an arbitrary unit scale. The concentration of anti-N that produced half of the maximal absorbance at 492 nm, given by the saturating concentration of anti-N in the cell ELISA plate, was arbitrarily defined as 1 U/ml [7].

### Statistical analysis

Differences in CSF anti-P_C22_, anti-whole P, anti-P_EX.C22_, and anti-C22-depleted rP0 among various groups were analyzed by Kruskal-Wallis test with multiple comparison (Scheffe's method). The correlation of anti-P_C22 _levels with anti-P_EX.C22 _or anti-C22-depleted rP0 levels and the correlation of anti-N levels with anti-P_C22_, anti-P_EX.C22_, or anti-C22-depleted rP0 levels were evaluated by Spearman rank correlation test. Differences in serum anti-P_C22_, anti-whole P, and anti-P_EX.C22 _levels between non-CNS SLE and NP-SLE were analyzed by Welch's *t *test.

## Results

**Figure 1:**
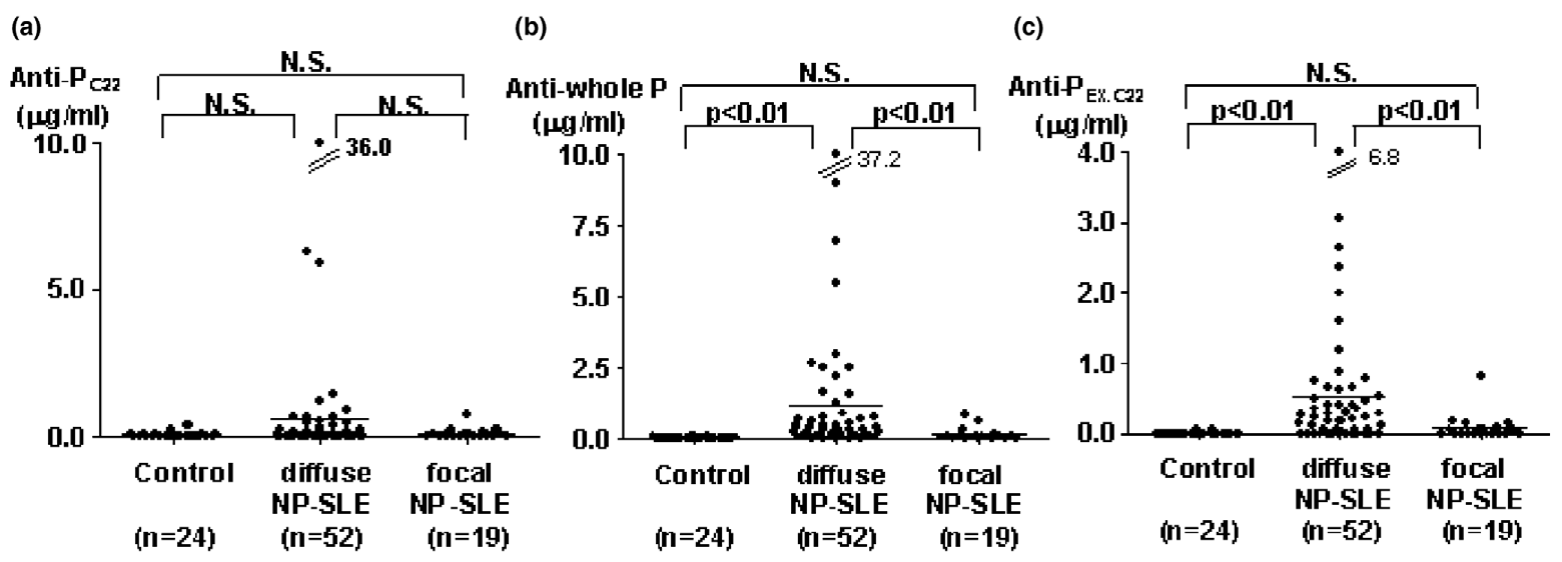
Cerebrospinal fluid antibodies to various components of ribosomal P proteins. CSF antibodies to the C-terminal 22-amino acid sequence of ribosomal P protein (anti-P_C22_), highly purified ribosomal P proteins (anti-whole P), and epitopes other than the C-terminal 22-amino acid sequence (anti-P_EX.C22_). Anti-P_C22 _**(a)**, anti-whole P **(b)**, and anti-P_EX.C22 _**(c) **in CSF from patients with non-inflammatory neurological diseases (Control), with diffuse neuropsychiatric systemic lupus erythematosus (NP-SLE), or with focal NP-SLE were compared. Horizontal lines indicate the mean values. Statistical analysis was performed by Kruskal-Wallis test with multiple comparisons (Scheffé's method). CSF, cerebrospinal fluid; N.S., not significant.

Initial experiments examined CSF anti-P_C22 _levels in the three groups of patients. Although anti-P_C22 _levels in CSF appeared to be higher in diffuse NP-SLE, there were no significant differences in their levels among the three groups, including diffuse NP-SLE, focal NP-SLE, and non-inflammatory neurological control (Figure 1a). The results therefore confirm the previous observation that CSF anti-P_C22 _might not be prevalent in diffuse NP-SLE. By contrast, anti-whole P levels in CSF from patients with diffuse NP-SLE were significantly elevated compared with those from patients with focal NP-SLE or with non-inflammatory neurological diseases (Figure 1b). In addition, it should be noted that CSF anti-whole P levels were significantly higher than CSF anti-P_C22 _levels in 67 patients with diffuse NP-SLE and focal NP-SLE (*P *< 0.0001 as evaluated by Wilcoxon signed rank test). These results suggest that in addition to anti-P_C22_, CSF from patients with NP-SLE might contain autoantibodies that recognize ribosomal P protein epitopes other than the C-terminal 22-amino acid sequence.

**Figure 2:**
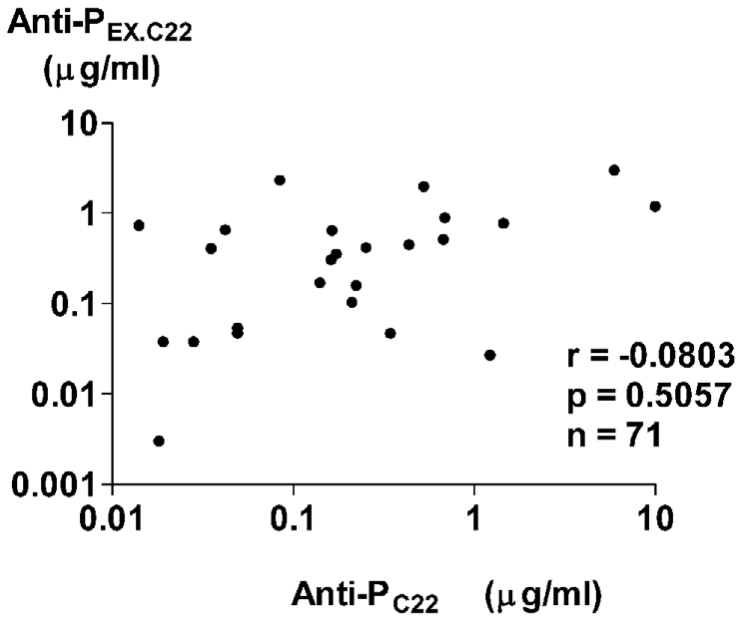
Correlation between autoantibodies to various components of ribosomal P proteins. The correlation between antibodies to the C-terminal 22-amino acid sequence of ribosomal P protein (anti-P_C22_) and those to the ribosomal P protein epitopes other than the C-terminal 22-amino acid sequence (anti-P_EX.C22_) in cerebrospinal fluid from patients with systemic lupus erythematosus (SLE), including 52 patients with diffuse neuropsychiatric SLE (NP-SLE) and 19 patients with focal NP-SLE, was analyzed. Statistical analysis was performed by Spearman rank correlation test.

To explore in detail the prevalence of the autoantibodies directed against the ribosomal P protein, epitopes other than the C-terminal 22-amino acid sequence (anti-P_EX.C22_) were calculated by subtracting anti-P_C22 _from anti-whole P. As can be seen in Figure 1c, anti-P_EX.C22 _levels in CSF from patients with diffuse NP-SLE were significantly elevated compared with those from patients with focal NP-SLE or with non-inflammatory neurological diseases. As shown in Figure 2, there was no significant correlation between CSF anti-P_C22 _and CSF anti-P_EX.C22 _levels, obviating the possibility that CSF anti-P_EX.C22 _activities might result from contamination of CSF anti-P_C22 _in patients with SLE. These results indicate that autoantibodies directed against ribosomal P protein epitopes other than the C-terminal 22-amino acid sequence are strongly associated with the development of diffuse NP-SLE. Moreover, the data indicate that the expression of such autoantibodies in CSF is not related to the presence of anti-P_C22 _in CSF.

**Figure 3:**
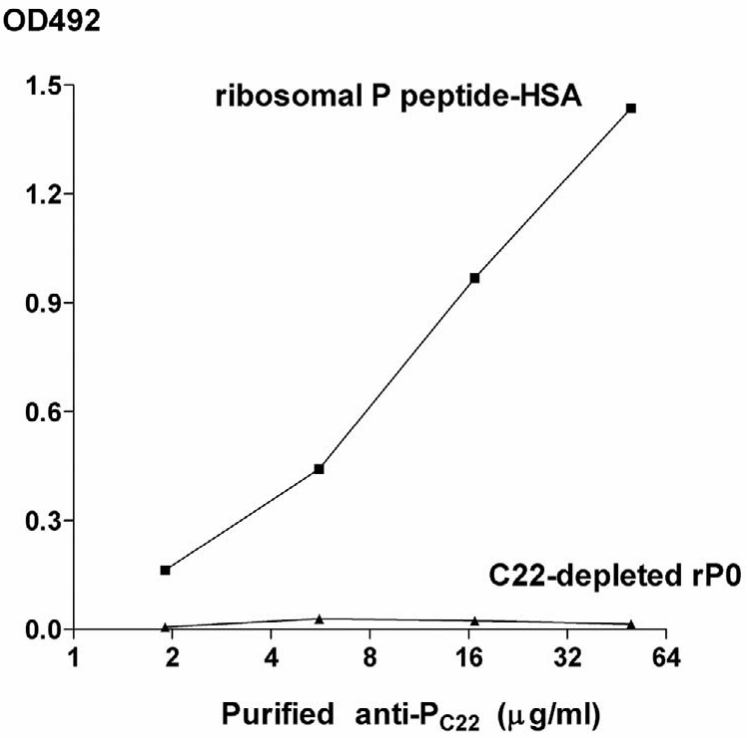
Differential reactivity of purified antibodies to the C-terminal 22 amino acids of ribosomal P protein. Differential reactivity of purified antibodies to the C-terminal 22-amino acid sequence of ribosomal P protein (anti-P_C22_) with ribosomal P peptide-human serum albumin (HSA) conjugates and with recombinant ribosomal P0 protein lacking the C-terminal 22-amino acid sequence (C22-depleted rP0). Purified anti-P_C22 _react with ribosomal P peptide-HSA conjugates, but not with C22-depleted rP0 on enzyme-linked immunosorbent assay plates. OD492 (optical density at 492 nm) values that are subtracted by non-specific binding activities are plotted.

**Figure 4:**
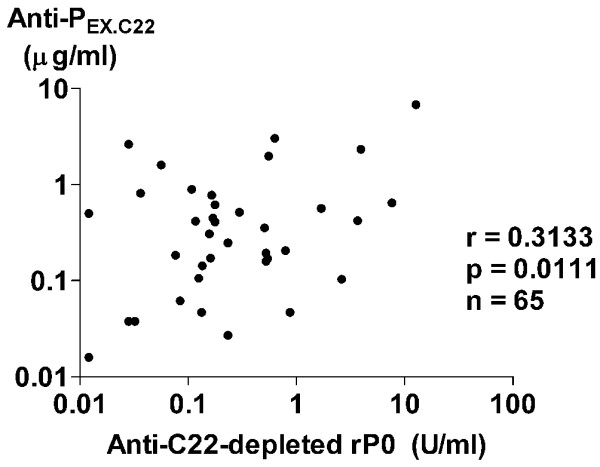
Correlation between autoantibodies to various components of ribosomal P proteins. The correlation between antibodies to recombinant ribosomal P0 protein lacking the C-terminal 22-amino acid sequence (anti-C22-depleted rP0) and those to the ribosomal P protein epitopes other than the C-terminal 22-amino acid sequence (anti-P_EX.C22_) in cerebrospinal fluid patients, including 47 patients with diffuse neuropsychiatric systemic lupus erythematosus (NP-SLE) and 18 patients with focal NP-SLE, was analyzed. Statistical analysis was performed by Spearman rank correlation test.

**Figure 5:**
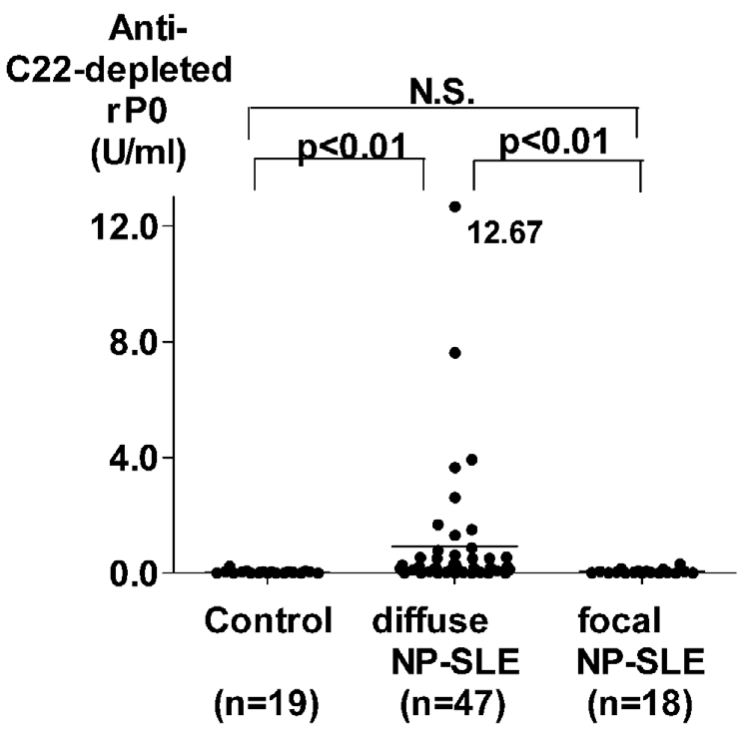
Cerebrospinal fluid antibodies to recombinant ribosomal P0 protein lacking the C-terminal 22-amino acid sequence. Antibodies to recombinant ribosomal P0 protein lacking the C-terminal 22-amino acid sequence (anti-C22-depleted rP0) (U/ml) in cerebrospinal fluid from patients with non-inflammatory neurological diseases (Control), with diffuse neuropsychiatric systemic lupus erythematosus (NP-SLE), or with focal NP-SLE were compared. Horizontal lines indicate the mean values. Statistical analysis was performed by Kruskal-Wallis test with multiple comparisons (Scheffé's method). N.S., not significant.

To confirm the presence of autoantibodies to ribosomal P protein epitopes other than the C-terminal 22-amino acid sequence, IgG antibodies to recombinant ribosomal P0 protein lacking the C-terminal 22 amino acids (C22-depleted rP0) were examined in CSF from 65 SLE patients with neuropsychiatric manifestations. Affinity-purified anti-P_C22 _reacted with ribosomal P peptide-HSA conjugates, but not with C22-depleted rP0, confirming the lack of the C-terminal 22-amino acid sequence in the C22-depleted rP0 (Figure 3). As shown in Figure 4, CSF anti-C22-depleted rP0 levels were significantly correlated with CSF anti-P_EX.C22 _levels in these 65 patients. In addition, anti-C22-depleted rP0 levels in CSF from patients with diffuse NP-SLE were significantly elevated compared with those from patients with focal NP-SLE or with non-inflammatory neurological diseases (Figure 5). Accordingly, the frequency of positive expression of anti-C22-depleted rP0 in CSF from patients with diffuse NP-SLE was higher than that in CSF from patients with focal NP-SLE or with non-inflammatory neurological diseases (Table 2). These results confirm the presence of autoantibodies to ribosomal P protein epitopes other than the C-terminal 22-amino acid sequence.

**Table 2:** Summary of the frequency of positive expression of antibodies to various ribosomal P protein components in cerebrospinal fluid^a^

	Percentage positive^b^		
	
	Control	Diffuse NP-SLE	Focal NP-SLE
Anti-P_C22_	4.2% (1/24)	23.1% (12/52)	5.3% (1/19)
Anti-whole P	0% (0/24)	78.8% (41/52)	31.6% (6/19)
Anti-P_EX.C22_	4.2% (1/24)	65.4% (34/52)	26.3% (5/19)
Anti-C22-depleted rP0	5.3% (1/19)	44.7% (21/47)	5.6% (1/18)

^a^Antibodies to the C-terminal 22-amino acid sequence of ribosomal P protein (anti-P_C22_), to highly purified ribosomal P proteins (anti-whole P), to the epitopes other than the C-terminal 22-amino acid sequence (anti-P_EX.C22_), and to recombinant ribosomal P0 protein lacking the C-terminal 22-amino acid sequence (anti-C22-depleted rP0) in cerebrospinal fluid from patients with non-inflammatory neurological diseases (Control), with diffuse neuropsychiatric systemic lupus erythematosus (NP-SLE), or with focal NP-SLE were compared. ^b^Cutoff values were set as the mean + 3 standard deviations of the values in control group. Values in parenthesis mean (numbers of patients with positive results/total patient numbers) in each group.

**Figure 6:**
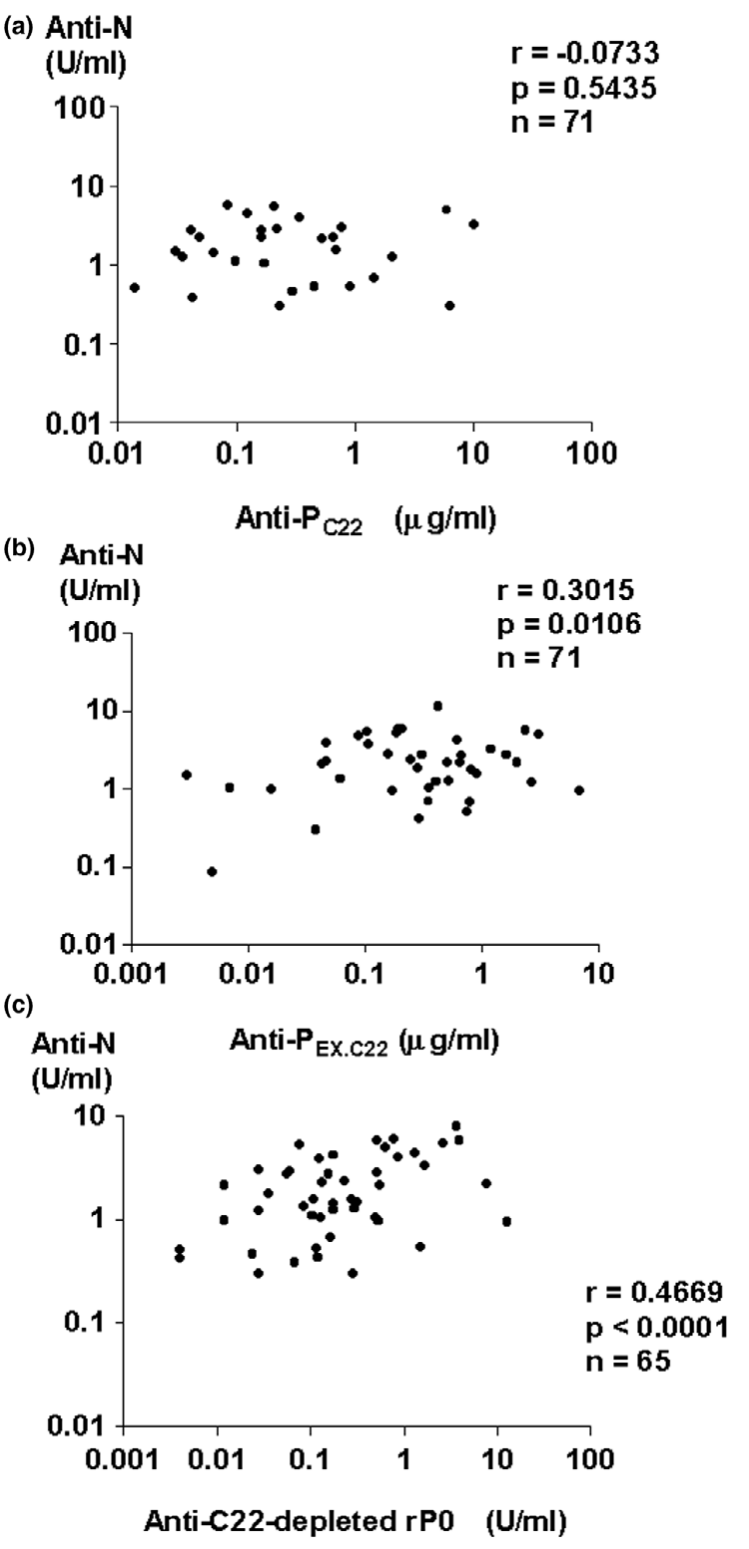
Correlation between autoantibodies to ribosomal P proteins and anti-neuronal cell antibodies. The correlation of antibodies to the C-terminal 22-amino acid sequence of ribosomal P proteins (anti-P_C22_) **(a)**, those to the ribosomal P protein epitopes other than the C-terminal 22-amino acid sequence (anti-P_EX.C22_) **(b)**, or those to recombinant ribosomal P0 protein lacking the C-terminal 22-amino acid sequence (anti-C22-depleted rP0) **(c) **with anti-neuronal cell antibodies (anti-N) in cerebrospinal fluid from systemic lupus erythematosus (SLE) patients, including 52 patients **(a,b) **or 47 patients **(c) **with diffuse neuropsychiatric SLE (NP-SLE) and 19 patients **(a,b) **or 18 patients **(c) **with focal NP-SLE, was analyzed. Statistical analysis was performed by Spearman rank correlation test.

We next examined whether CSF anti-whole P might account for anti-N activities in CSF from patients with NP-SLE. As shown in Table 3, levels of CSF anti-whole P and anti-P_C22 _as well as CSF anti-N were decreased when CSF was incubated with paraformaldehyde-fixed SK-N-MC cells for 120 minutes at room temperature, confirming that CSF anti-whole P or anti-P_C22 _are constituents of CSF anti-N. However, as shown in Figure 6a, CSF anti-N levels were not significantly correlated with CSF anti-P_C22 _levels in SLE patients, including those with diffuse NP-SLE and focal NP-SLE. By contrast, CSF anti-N levels were significantly correlated with CSF anti-P_EX.C22 _or CSF anti-C22-depleted rP0 levels (Figure 6b,c). The data therefore suggest that C22-depleted rP0 might contain one of the major targets, against which CSF anti-N are directed.

**Table 3:** Absorption of CSF autoantibodies to various components of ribosomal P proteins by neuronal cells

Patient	Autoantibodies	Without absorption	With absorption
1	Anti-whole P (μg/ml)	7.243	1.435
	Anti-P_C22 _(μg/ml)	3.456	1.019
	Anti-P_EX.C22 _(μg/ml)	3.787	0.416
	Anti-N (U/ml)	4.083	1.950
2	Anti-whole P (μg/ml)	0.140	0.050
	Anti-P_C22 _(μg/ml)	0.070	0.042
	Anti-P_EX.C22 _(μg/ml)	0.070	0.008
	Anti-N (U/ml)	0.789	0.588

Cerebrospinal fluid (CSF) samples (50 μl/well) were incubated in wells of a 96-well flat-bottomed microtiter plate with or without confluent SK-N-MC cells fixed with 1% paraformaldehyde at room temperature for 2 hours. After the incubation, CSF samples were recovered and were examined for anti-whole P, anti-P_C22_, anti-P_EX.C22_, and anti-N as described in Materials and methods. Anti-N, anti-neuronal cell antibodies; anti-P_C22_, antibodies directed against the C-terminal 22-amino acid sequences of ribosomal P protein; anti-P_EX.C22_, autoantibodies directed against the ribosomal P protein epitopes other than the C-terminal 22-amino acid sequence; anti-whole P, antibodies to the whole ribosomal P proteins.

**Figure 7:**
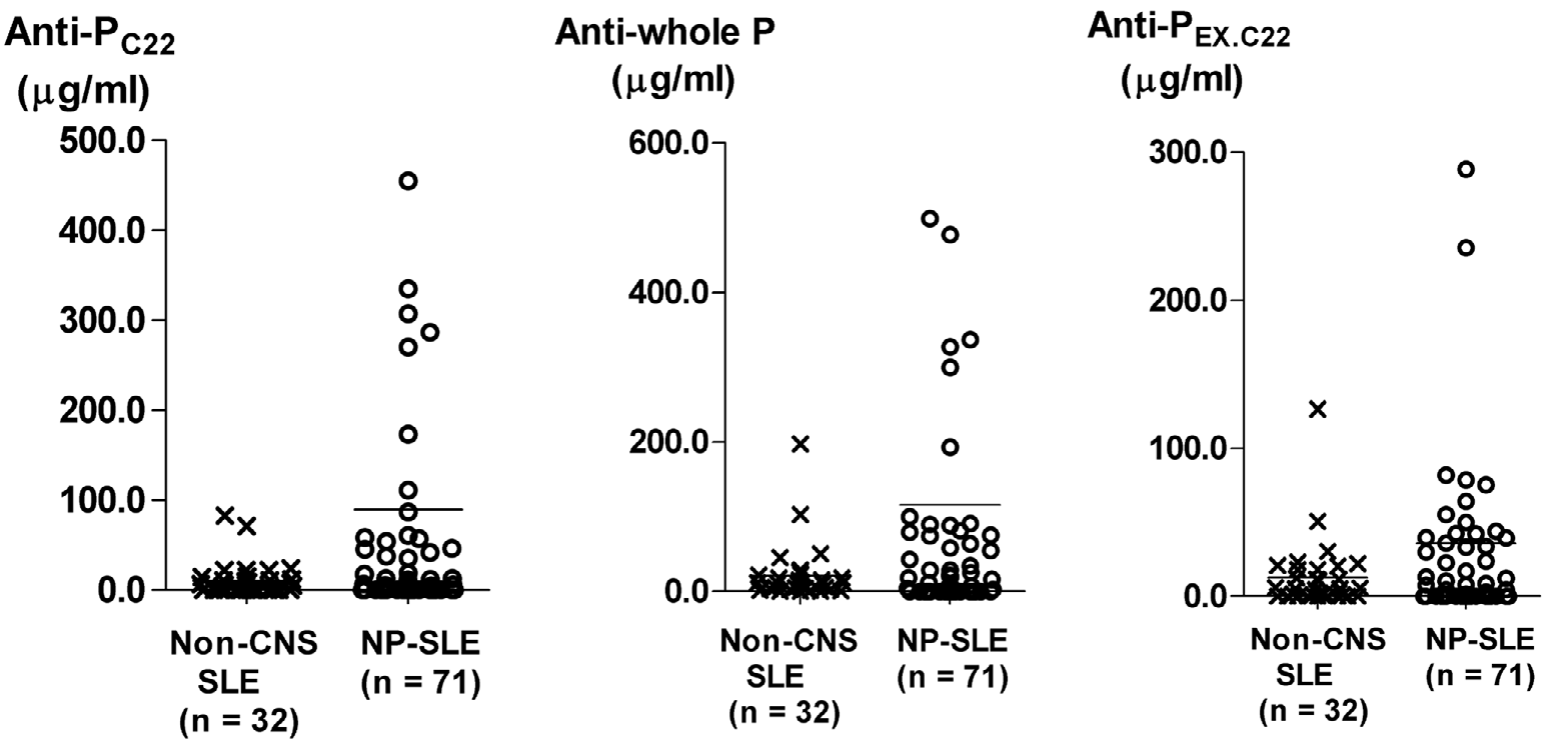
Serum autoantibodies to various components of ribosomal P proteins. Anti-P_C22_, anti-whole P, and anti-P_EX.C22 _in sera from SLE patients without neuropsychiatric manifestations (non-CNS SLE) (cross), with diffuse NP-SLE (open circle), or with focal NP-SLE (closed circle) were compared. Horizontal lines indicate the mean values. Statistical analysis between non-CNS SLE versus NP-SLE (focal + diffuse) was performed by Welch's *t *test. Anti-P_C22_, antibodies directed against the C-terminal 22-amino acid sequences of ribosomal P protein; anti-P_EX.C22_, autoantibodies directed against the ribosomal P protein epitopes other than the C-terminal 22-amino acid sequence; anti-whole P, antibodies to the whole ribosomal P proteins; non-CNS SLE, systemic lupus erythematosus without neuropsychiatric manifestations; NP-SLE, neuropsychiatric systemic lupus erythematosus; SLE, systemic lupus erythematosus.

Finally, we examined serum levels of anti-P_C22_, anti-whole P, and anti-P_EX.C22 _in patients with non-CNS SLE or with NP-SLE. The values of anti-P_C22_, anti-whole P, and anti-P_EX.C22 _in 24 patients with non-SLE non-inflammatory neurological diseases were 2.44 ± 2.92 μg/ml, 4.92 ± 6.51 μg/ml, and 3.41 ± 6.06 μg/ml (mean ± standard deviation), respectively. As shown in Figure 7, serum anti-P_C22 _as well as anti-whole P levels in NP-SLE were significantly elevated compared with those in non-CNS SLE, which is consistent with previous studies [3-5]. Serum anti-P_C22 _and anti-whole P levels appeared to be higher in diffuse NP-SLE than those in focal NP-SLE, although there were no statistical significances by Kruskal-Wallis test with multiple comparisons. Of note, there were no significant differences in serum anti-P_EX.C22 _levels between non-CNS SLE and NP-SLE. These results suggest that in contrast with the CSF results, serum anti-P_C22_, but not serum anti-P_EX.C22_, are associated with NP-SLE, especially diffuse NP-SLE.

## Discussion

A number of studies have suggested that CSF anti-N play an important role in the pathogenesis of diffuse NP-SLE [7,11]. However, the epitopes to which CSF anti-N are directed have not been delineated. Of note, previous studies have demonstrated that epitopes antigenically related to ribosomal P proteins are present on the surface of SK-N-MC neuroblastoma cells [12]. Although anti-P_C22 _have been shown to be major autoantibodies to ribosomal P proteins [3,4,22], the frequency of their detection in CSF from patients with diffuse NP-SLE was not high enough to ensure their involvement in the pathogenesis of this disease [3,4,7]. Therefore, it was suggested that anti-P_C22 _might not be a major constituent of anti-N in CSF from patients with diffuse NP-SLE. Consistently, the data in the current studies indicated that CSF anti-P_C22 _levels were not significantly elevated in patients with diffuse NP-SLE compared with those in patients with focal NP-SLE or with non-inflammatory neurological diseases. However, it was still possible that CSF autoantibodies directed to ribosomal P protein epitopes other than the C-terminal 22-amino acid sequence were more prevalent. Thus, the results in the current studies have also demonstrated that levels of CSF anti-whole P as well as CSF anti-P_EX.C22 _were significantly higher in patients with diffuse NP-SLE than in patients with focal NP-SLE or non-inflammatory neurological diseases. The data therefore indicate that CSF antibodies to ribosomal P protein epitopes other than the C-terminal 22-amino acid sequence are associated with diffuse NP-SLE.

To confirm the presence of antibodies for the epitopes representing regions of the ribosomal P proteins other than the C-terminal 22-amino acid sequence, antibodies to recombinant ribosomal P0 protein lacking the C-terminal 22 amino acids (C22-depleted rP0) [21] were evaluated. The results clearly demonstrate that CSF anti-C22-depleted rP0 levels were significantly correlated with CSF anti-P_EX.C22 _levels. In addition, levels of CSF anti-C22-depleted rP0 as well as CSF anti-P_EX.C22 _were significantly elevated in diffuse NP-SLE. The data therefore confirm that CSF antibodies to ribosomal P protein epitopes other than the C-terminal 22-amino acid sequence play a role in the pathogenesis of diffuse NP-SLE, but further studies are required to identify the fine epitopes.

In has been demonstrated that purified human plasma membranes contain a 38-kDa protein that is closely related or identical to ribosomal P0 proteins [12]. Therefore, it was suggested that autoantibodies to ribosomal P proteins, especially those directed to epitopes other than the C-terminal 22-amino acid sequence, might be involved (at least in part) in CSF anti-N activities. In fact, levels of CSF anti-P_EX.C22 _as well as CSF anti-P_C22 _or CSF anti-whole P were decreased after incubation of CSF with paraformaldehyde-fixed SK-N-MC cells, confirming that CSF anti-P_EX.C22 _as well as anti-P_C22 _are constituents of CSF anti-N. However, CSF anti-P_C22 _levels were not significantly correlated with CSF anti-N levels in the present study. By contrast, CSF anti-P_EX.C22 _or CSF anti-C22-depleted rP0 levels were significantly correlated with CSF anti-N levels. These results indicate that ribosomal P0 proteins contain one of the major targets of CSF anti-N in their portions other than the C-terminal 22-amino acid sequence. Of note, recent studies have demonstrated that autoantibodies directed against the *N*-methyl-d-aspartate (NMDA) receptor mediated apoptotic death of neurons *in vivo *and *in vitro *in murine systems [23]. Of note, anti-NMDA receptor antibodies were also detected in CSF from a single patient with SLE [22]. It is therefore likely that anti-NMDA receptor antibodies might also be involved in CSF anti-N activities and thus play a pivotal role in the pathogenesis of diffuse NP-SLE. Further studies with a large number of patients are required to confirm the involvement of anti-NMDA receptor antibodies in diffuse NP-SLE and to explore its relationship with anti-N.

A number of studies have indicated that serum anti-ribosomal P protein antibodies, including anti-P_C22 _or anti-whole P, are frequently observed in patients with NP-SLE [3-5,24]. Consistently, the results in the current studies have also disclosed that levels of serum anti-P_C22 _as well as serum anti-whole P are significantly higher in NP-SLE than those in non-CNS SLE. Of note, serum anti-P_EX.C22 _levels were not significantly elevated in NP-SLE compared with those in non-CNS SLE. These findings contrast sharply with the results of CSF studies. Thus, in CSF, anti-P_EX.C22_, but not anti-P_C22_, were significantly associated with diffuse NP-SLE, whereas in serum, anti-P_C22_, but not anti-P_EX.C22_, were associated with NP-SLE.

The mechanism by which anti-whole P cause neuronal damage remains unclear. We previously reported that the expression of IL-6 mRNA in neurons was upregulated in the brain of an SLE patient who died of active diffuse NP-SLE [25]. Of note, we recently disclosed that anti-P_C22 _upregulate the expression of mRNAs for IL-6 and tumor necrosis factor-alpha in human peripheral blood monocytes [20]. It should be pointed out that anti-P_EX.C22 _as well as anti-P_C22 _might be able to bind the ribosomal P protein on neuronal cells [12]. Taken together, these results suggest that anti-whole P or anti-P_EX.C22 _might also upregulate the expression of IL-6 mRNA in neurons and thus result in the alteration of their functions. Further studies to explore the targets and the effects on their functions of anti-P_C22 _and anti-P_EX.C22 _(or anti-P_AA9_) would improve our understanding of the pathogenesis of NP-SLE.

In summary, the current studies have demonstrated that the expression of autoantibodies directed against the epitopes of ribosomal P proteins other than the C-terminal 22-amino acid sequence is increased in CSF from patients with diffuse NP-SLE. The presence of such autoantibodies might account for CSF anti-N activities, although there might be other antibodies that bind to neuronal cells, such as anti-NMDA receptor antibodies. Further studies to explore the whole spectrum of epitopes of neurons to which autoantibodies are directed as well as the mechanism by which such autoantibodies cause damage to neurons are needed for a complete understanding of the pathogenesis of diffuse NP-SLE.

## Conclusion

The present study has disclosed that CSF IgG antibodies to the epitopes of ribosomal P0 proteins other than the C-terminal 22 amino acids are associated with the development of diffuse NP-SLE as one of the major CSF anti-N components.

## Abbreviations

ACR = American College of Rheumatology; anti-C22-depleted rP0 = antibodies directed against recombinant ribosomal P0 protein lacking the C-terminal 22 amino acids; anti-N = anti-neuronal cell antibodies; anti-P_C22 _= antibodies directed against the C-terminal 22-amino acid sequences of ribosomal P protein; anti-P_EX.C22 _= autoantibodies directed against the ribosomal P protein epitopes other than the C-terminal 22-amino acid sequence; anti-whole P = antibodies to the whole ribosomal P proteins; C22-depleted rP0 = recombinant ribosomal P0 fusion protein lacking the C-terminal 22 amino acids; CNS = central nervous system; CSF = cerebrospinal fluid; ELISA = enzyme-linked immunosorbent assay; HSA = human serum albumin; IgG = immunoglobulin G; IL-6 = interleukin-6; NMDA = *N*-methyl-d-aspartate; non-CNS SLE = systemic lupus erythematosus without neuropsychiatric manifestations; NP-SLE = neuropsychiatric systemic lupus erythematosus; OD_492 _= optical density at 492 nm; PBS = phosphate-buffered saline; SLE = systemic lupus erythematosus.

## Competing interests

The authors declare that they have no competing interests.

## Authors' contributions

SH designed the study and participated in experimental procedures, collection, analysis, and interpretation of data and manuscript preparation. YA and MT contributed to the collection and analysis of data. TY helped to prepare C22-depleted rP0 and to develop ELISA for anti-C22-depleted rP0. All authors read and approved the final text before submission of the manuscript.
